# Molecular Aspects of Regeneration Mechanisms in Holothurians

**DOI:** 10.3390/genes12020250

**Published:** 2021-02-10

**Authors:** Igor Yu. Dolmatov

**Affiliations:** A.V. Zhirmunsky National Scientifc Center of Marine Biology, Far Eastern Branch, Russian Academy of Sciences, Palchevsky 17, 690041 Vladivostok, Russia; idolmatov@mail.ru

**Keywords:** holothurians, regeneration, asexual reproduction, gene expression, immunity response, extracellular matrix remodeling, Wnt signaling, dedifferentiation, transdifferentiation

## Abstract

Holothurians, or sea cucumbers, belong to the phylum Echinodermata. They show good regenerative abilities. The present review provides an analysis of available data on the molecular aspects of regeneration mechanisms in holothurians. The genes and signaling pathways activated during the asexual reproduction and the formation of the anterior and posterior parts of the body, as well as the molecular mechanisms that provide regeneration of the nervous and digestive systems, are considered here. Damage causes a strong stress response, the signs of which are recorded even at late regeneration stages. In holothurian tissues, the concentrations of reactive oxygen species and antioxidant enzymes increase. Furthermore, the cellular and humoral components of the immune system are activated. Extracellular matrix remodeling and Wnt signaling play a major role in the regeneration in holothurians. All available morphological and molecular data show that the dedifferentiation of specialized cells in the remnant of the organ and the epithelial morphogenesis constitute the basis of regeneration in holothurians. However, depending on the type of damage, the mechanisms of regeneration may differ significantly in the spatial organization of regeneration process, the involvement of different cell types, and the depth of reprogramming of their genome (dedifferentiation or transdifferentiation).

## 1. Introduction

Holothurians, or sea cucumbers, are members of the class Holothuroidea, phylum Echinodermata. They have an elongated, often worm-like, body bearing various outgrowths. Like all echinoderms, holothurians are exclusively marine animals. They live in a wide range of depths, from shallow, intertidal zones to a depth of 5000 m or more, in all regions of the world’s oceans. Most holothurians are benthic organisms, but there are also swimming species [1,2], and, possibly, fully pelagic ones [3]. These animals are of great importance as focus species of fisheries and aquaculture in South-East Asia and Australia, where about 47 holothurian species are used for various purposes [4,5].

Holothurians have good regenerative abilities [6,7]. They can heal cutaneous wounds and regenerate small appendages of the body such as tentacles and tube feet. Holothurians are able to restore all internal organs, including the gonad [7,8,9,10,11,12]. Moreover, they can regenerate large regions of the body after transverse fission or cutting into two or three parts [8,13,14,15,16,17].

Investigations into regeneration in holothurians are most frequently associated with studies on restoration of their internal organs, since these animals possess a unique type of autotomy referred to as evisceration. Holothurians can eject a part of internal organs (viscera) in case of deterioration of environmental conditions (increase in water temperature, freshening, or pollution) or due to various artificial impacts (weak electric current, injection of distilled water, KCl, or dilute ammonia solution into the body cavity) [13]. In a number of holothurian species, evisceration is seasonal [18,19,20]. The function and causes of evisceration still remain poorly understood. The ejection of viscera can be provoked by a predator’s attack, invasion and reproduction of parasites, or accumulation of waste products in tissues [19]. Moreover, evisceration can be a way to survive adverse conditions. For example, holothurians often eviscerate in response to deteriorating environmental conditions such as reduction in oxygen concentration, water pollution, and temperature rise [21,22].

Viscera can be removed in two different ways: Through the anterior or posterior ends of the body. During evisceration through the anal orifice (posterior evisceration), the middle part of the digestive tube located between the esophagus and the cloaca, the respiratory organs (respiratory trees), and part of the reproductive system (gonadal tubules) are removed [13,22]. This way is typical mainly of holothurians from the orders Holothuriida and Synallactida, as well as of a number of species from the order Dendrochirotida [8,22,23]. Evisceration through the anterior end of the body (anterior evisceration) occurs only in members of the order Dendrochirotida [8,13,22]. In this case, the entire digestive tract is removed, except the cloaca, the oral complex of organs (aquapharyngeal bulb, AB), and part of the gonadal tubules.

When considering regeneration in holothurians, one characteristic feature of echinoderms deserves particular mentioning. To date, no reliable evidence for the presence of stem cells in these animals have been found [24,25]. The exceptions are primordial germ cells and, probably, coelomocyte stem cells [26,27]. All attempts to detect stem cells using various mammalian stem cell markers such as Yamanaka factors or mammalian intestinal stem cell markers have failed so far [28,29,30]. The fact that pluripotency factors may be involved not only in maintaining the undifferentiated state of stem cells, but also in dedifferentiation and transdifferentiation of specialized cells also hampers the search [31,32,33].

On the other hand, numerous morphological studies show that regeneration in echinoderms occur due to differentiated cells of organ remnant [7,9,10,12,34,35,36,37,38,39,40,41]. The good regenerative abilities in echinoderms are explained by the ease of dedifferentiation and transdifferentiation of specialized cells [9,36,42,43,44,45].

Coelomic epithelium is a special tissue system of echinoderms that plays an important role in regeneration. It has been shown that coelomic epithelial cells in holothurians and crinoids can be involved in the formation of luminal epithelium of the gut [46,47,48]; in holothurians and sea urchins, it is involved in muscle development and regeneration [9,34,49,50,51]. Its cells are first to respond to damage, undergoing dedifferentiation and beginning to migrate and proliferate [42,43,52,53,54]. Thus, coelomic epithelium in echinoderms is a system with quite substantial histoblastic potencies [7]. Coelomic epithelium in vertebrates performs similar functions. It is a main source of many cell types, mainly fibroblasts and smooth muscle, but also organ-specific cells with very specialized physiological functions [55].

Coelomic epithelium is a derivative of mesoderm and is formed by the growth of the third pair of coelomes, somatocoels [56,57]. In holothurians, this process causes the formation of a large body cavity filled with coelomic fluid. The somatocoel wall forms coelomic epithelium (mesothelium) that covers all the internal organs. Thus, coelomic epithelia of different organs have a common origin. However, mesothelium is modified in the process of organogenesis and is represented in various organs by different morphological varieties. The latter differ both in the structure of epithelial cells (peritoneocytes and myoepithelial cells) and in the function performed [56]. The mesothelium also includes nerve cells and axon bundles forming the basiepithelial nerve plexus [56]. The final stage of the coelomic epithelium development is the formation of myocytes and muscle bundles that make up large muscles (longitudinal muscle bands, retractor muscles, etc.) [49,50,51]. 

Another derivative of somatocoels is gut mesentery. It is formed through merger of adjacent regions of the walls of the left and right somatocoels on the dorsal and ventral sides of holothurians [56,57]. A layer of connective tissue is formed between the walls of the coelomes, which leads to the formation of gut mesentery supporting the gut in the body cavity. Subsequently, the ventral mesentery is destroyed.

To date, regeneration has been described in sufficient detail from four holothurian species belonging to three orders: Synallactida (*Apostichopus japonicus* (Selenka, 1867)), Holothuriida (*Holothuria* (*Selenkothuria*) *glaberrima* Selenka, 1867), and Dendrochirotida (*Eupentacta fraudatrix* (D’yakonov and Baranova in D’yakonov, Baranova and Savel’eva, 1958) and *Cladolabes schmeltzii* (Ludwig, 1875)). Morphological events of regeneration in them were studied in detail, morphogenesis was described on the cellular level, and sources of regeneration of various organs were identified, which helps interpret molecular genetic data. Furthermore, each of the holothurian species above has its own characteristics of regeneration, which allows a more comprehensive study of the regeneration phenomenon [58]. *C. schmeltzii* has the capability of asexual reproduction by transverse fission and, accordingly, regeneration of body fragments [23,45,59,60]. *A. japonicus* and *H. glaberrima* eviscerate the internal organs through the anal orifice, and both species lose the same organs [37,61]. After this type of damage, two gut anlagen are formed. Luminal epithelium of the gut regenerated from the epithelia of cloaca (posterior anlage) and esophagus remnant (anterior anlage). Studies have shown that gut regeneration is also very similar on the cellular level in *A. japonicus* and *H. glaberrima* [37,41,61,62]. The holothurian *E. fraudatrix* is capable of anterior evisceration, while the gut regeneration in this species is radically different from that in the other above-listed species [42,61]. The major distinguishing feature of the digestive system regeneration in *E. fraudatrix* is the formation of enterocytes through the transdifferentiation of coelomic epithelial cells [46].

## 2. Regeneration after Fission

### 2.1. Morphological Aspects of Fission and Regeneration

Echinoderms exhibit different ways of asexual reproduction, of which the most common one is fission [63]. To date, 16 species of fissiparous holothurians are known that can be model objects for studying the mechanisms of fission with subsequent regeneration [59]. Most holothurian species have a thick body wall composed of connective tissue [56]. Accordingly, ECM remodeling is an important component of the transverse fission mechanism [63,64,65]. It has been shown that connective tissue in holothurians possesses a unique ability to alter its mechanical properties under effects of various factors [66,67]. Thus it is called mutable collagenous tissue (MCT) [68] or catch connective tissue [69]. The body-wall connective tissue in holothurians can occur in three states: Stiff, standard, and soft [65]. The mechanical stretching results in the decreased stiffness of the connective tissue and promotes its transition into the soft state [65].

Furthermore, stiffness of MCT depends on the interaction of three protein groups: Matrix metalloproteinases (MMPs), tissue inhibitors of metalloproteinases (TIMPs), and cross-link complexes connecting collagen fibrils to one another [70]. As the activity of TIMPs increases, MMPs are blocked. As a result, cross-links are formed between collagen fibrils, and MCT strengthens. Conversely, an increase in the MMPs concentration or activity in connective tissue leads to the destruction of cross-link complexes. This destruction enables collagen fibrils to slide along one another, which brings MCT into a compliant state. Thus, a local change in properties of the body-wall connective tissue allows holothurian to divide into two parts.

**Figure 1:**
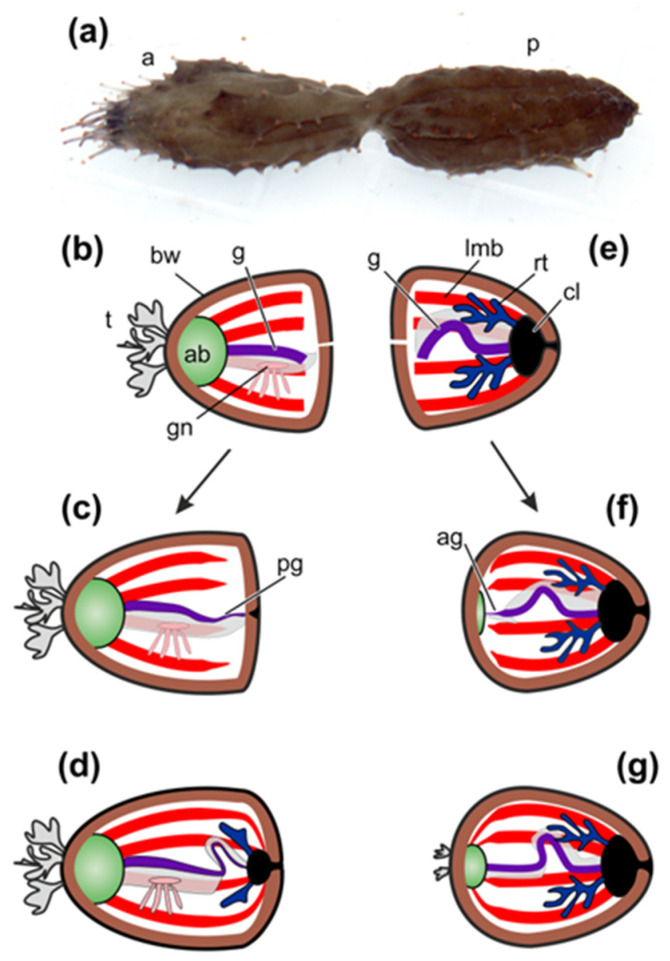
Scheme of regeneration of internal organs after fission in *Cladolabes schmeltzii*. (**a**) *C. schmeltzii* during fission. (**b**) Anterior fragment just after fission. (**c**) Formation of gut and cloaca in anterior fragment. (**d**) Formation of respiratory trees in anterior fragment. (**e**) Posterior fragment just after fission. (**f**) Formation of aquapharyngeal bulb (AB) and gut anlage in posterior fragment. (**g**) Posterior fragment with regenerated internal organs. a, anterior part; ab, aquapharyngeal bulb; ag, anterior anlage of gut; bw, body wall; cl, cloaca; g, gut; gn, gonad; lmb, longitudinal muscle band; p, posterior part; pg, posterior anlage of gut; rt, respiratory tree; t, tentacles.

After division the anterior fragment of holothurian contains the AB, the gonad, and the anterior half of the intestinal tube (Figure 1a,b). The posterior part of the gut, the cloaca, and the respiratory organs (respiratory trees) remain in the posterior fragment (Figure 1a,e).

The first morphological signs of regeneration in both fragments are detected after 5 days post-fission [45,60]. In the anterior fragment of holothurians, the remaining part of the gut becomes slightly shortened, probably due to the destruction of a portion of cells, and its end region is narrowed. A connective-tissue thickening grows out from the gut along the edge of the gut mesentery and gradually extends posteriorly [45]. Simultaneously with this process, enterocytes in the wound zone begin to dedifferentiate and divide mitotically. Intercellular junctions between them are not broken. The luminal epithelium of the gut grows into the connective-tissue thickening (Figure 1c). Subsequently, the anlage gradually extends backwards and reaches the posterior wall of the body, where the cloaca and respiratory trees have already formed by this time (Figure 1d) [45]. 

In the posterior fragment, regeneration begins with the formation of AB, which develops at the anterior end of the animal [60]. The gut regeneration in the posterior fragment occurs through the transformation of the anterior part of the digestive tube. The anterior end of the gut remnant becomes thinner. It gives rise to a connective-tissue thickening, which grows anteriorly along the mesentery edge and connects to AB (Figure 1f). Enterocytes are dedifferentiated and divide mitotically, but intercellular junctions between them are not destroyed. As a result, the luminal epithelium of the gut grows into the connective-tissue thickening and then into AB. Subsequently, tentacles and organs of the water–vascular system are formed (Figure 1g). Thus, the main regeneration processes in holothurians after fission are ECM remodeling, cell dedifferentiation, cell proliferation, and epithelial morphogenesis. 

In regeneration, the significance of ECM remodeling is associated with the fact that most organs, both in holothurians and in all echinoderms, are epithelial formations (mostly tubular), containing a large amount of connective tissue. Accordingly, when organs regenerate after fission, the connective-tissue base (connective-tissue thickening) is formed first, and then certain cells or epithelia migrate into it [45,60]. This pattern of organ formation is typical of regeneration not only after fission, but also after evisceration (see below). For this reason, major attention was paid to the structure of connective tissue and the mechanisms of its modification during asexual reproduction in holothurians [71].

### 2.2. Structural Components of Connective Tissue

Transcripts of genes of many ECM components that are characteristic of most multicellular animals—collagens, proteoglycans, and glycoproteins—have been found in holothurians [71,72,73,74,75]. On the other hand, the differences in connective tissue of echinoderms and vertebrates have also been identified. In particular, one of the major ECM components in vertebrates is elastin, whose fibers are formed through the polymerization of tropoelastin [76]. Echinoderms lack the *tropoelastin* gene [71]. 

Another distinguishing feature of holothurian ECM is the absence of tenascins and fibronectins [77]. These proteins play an important role in the structural integrity of connective tissues in vertebrates [78,79]. In some of holothurians, transcripts have been found that are blasted as tenascin-like proteins [71,72,80]. These contigs encode the domains characteristic of tenascins, EGF, FBG, and TILa. Nevertheless, according to Hynes [81], all these domains are ancient in origin and are found in many animals; however, in the combination characteristic of tenascins, they occur only in chordates. No transcripts of the *fibronectin* gene were found in *C. schmeltzii* [71]. Thus, the lack of such proteins as elastin, fibronectin, and tenascin indicates significant differences in the connective tissue organization between echinoderms and chordates. 

Due to the lack of elastin, its function in echinoderms is probably performed by fibrillin. In ECM, it forms a network consisting of microfibrils 10–14 nm in diameter that surrounds and penetrates bundles of collagen fibrils [82]. It is suggested that fibrillin microfibrils may be involved in ligament contraction in sea urchins [83]. As has been shown recently, fibrillin microfibrils play an important role in ECM functioning [84]. They are involved in the distribution, accumulation, and modulation of signals from transforming growth factor-β (TGF-β) and bone morphogenetic protein (BMP) that regulate various aspects of cell activity, including ECM formation and remodeling [85]. In addition, fibrillins can bind to integrin receptors and a number of other molecules and, as a result, signals about changes in the extracellular microenvironment are transmitted to cell. In fact, fibrillin microfibrils form niches accumulating various factors [86]. 

Holothurians have *laminins*, *nidogens*, *fibulins*, *agrin*, *dystroglycan*, *perlecan*, and *thrombospondins*. The proteins encoded by these genes, along with collagens and fibrillins, are included in the basic set of “basement membrane ECM toolkit”, typical of all Bilateria [87]. The genes are involved in the construction and functioning of connective tissue. For example, fibulins can bind to many ECM components such as fibrillin, and play an important role in stabilizing supramolecular complexes of connective-tissue [88]. Fibulin 1 has been shown to accelerate the disintegrin and metalloproteinase with thrombospondin motifs (ADAMTS)-mediated aggrecan proteolysis and, thus, participate in tissue renewal [89]. 

Thrombospondins (TSPs) play certain role in the organization of ECM, since they can serve as molecular bridges between various components of connective tissue [90]. They have been shown to interact with MMPs, fibrillar collagen, and TGF-β. TSP-1 and TSP-2 can inhibit the activity of MMP2 [91] and regulate its level in extracellular matrix [92,93]. Thus, TSPs interacts with the molecules that in echinoderms may be involved in mechanisms responsible for modifying ECM during fission and regeneration.

### 2.3. Proteins Modifying ECM

In addition to the genes of connective-tissue structural components, holothurians have a wide variety of genes encoding ECM-modifying proteins. Many different serine, cysteine, aspartyl, and metal peptidases and their inhibitors have been identified [71,94,95,96,97,98]. In the study of regeneration in holothurians, much attention is paid to MMPs that can degrade collagen [96,97,99,100,101]. In the transcriptomes of holothurians, products of *MMPs* genes and genes of their inhibitors (*TIMPs*) have been found [71,72,75,102]. In some of holothurian species, the number of *TIMPs* can reach 45 [103]. The increase in the number of *TIMPs* occurred, apparently, due to the increased role of connective tissue in the vital activities of echinoderms such as asexual reproduction, autotomy, and regeneration [71,103]. 

One of the factors that have an effect on the connective tissue strength in echinoderms is assumed to be *tensilin* [67,104]. Tensilins are TIMP-like proteins [67,71]. It is worth mentioning that the *tensilin* gene is found only in species belonging to members of relatively young groups of holothurians and, consequently, have formed within the class Holothuroidea [71]. No similar proteins are found in Apodida (the most ancient order of holothurians [105]), as well as in other echinoderms. This may indicate evolutionary changes in the mechanisms of functioning of ECM that occurred in the phylogeny of echinoderms. Depending on needs, the properties and functions of TIMP-like proteins varied in different classes of Echinodermata. Holothurians differ from other echinoderms by their more developed body wall and the almost complete reduction of skeleton [56]. The increased role of connective tissue was probably accompanied by the modification of ECM remodeling mechanisms and the emergence of a separate group of TIMP-like genes, *tensilins*. 

### 2.4. Fission Mechanisms

The genes coding proteins of “basement membrane ECM toolkit” are expressed in dividing individuals of *C. schmeltzii* [71]. This indicates a large-scale rearrangement in epithelia during fission. Moreover, a comparison of intact and dividing *C. schmeltzii* has revealed some qualitative differences in the spectrum of proteinases and their inhibitors. In particular, *ADAMTS7*, *ADAMTS9*, *cathepsin D*, *serine proteases 1-3*, and *TIMP10* are activated in asexual reproduction [71]. The presence of transcripts of these genes in tissues of dividing individuals indicates the possible involvement of the respective proteins in the modification of ECM and the mechanisms of holothurians’ fission. At the same time, there are no differences in the qualitative composition of MMPs. Probably, if MMPs are involved in the asexual reproduction mechanisms, then the level of activity of proteinases, rather than their spectrum, varies in the case of fission. Similarly, there are no qualitative differences in the set of MMPs during regeneration of internal organs in holothurian *E. fraudatrix* [97,98]. 

### 2.5. Preparation for Whole Body Regeneration

Simultaneously with the division of holothurian’s body into anterior and posterior fragments, the internal organs are prepared for the subsequent regeneration. In *C. schmeltzii*, the Wnt and Sonic Hedgehog (Shh) signaling pathways are activated in asexual reproduction [71]. Transcripts of *Wnt2*, *Wnt4*, and *frizzled9/10* are found only in individuals that are in the process of fission and are absent from intact animals. Also, the gene that encodes smoothened, a receptor for the Shh signaling pathway, is active only during asexual reproduction. This may mean that the Wnt and Shh signaling pathways are involved in the regulation of fission and/or in the preparation for the subsequent regeneration of internal organs. 

In *C. schmeltzii* undergoing fission, transcripts of genes of 26 transcription factors (TFs) related to the morphogenesis regulation have been detected [71]. Many of them, such as *Tbx2/3*, *SoxD1*, *FoxK2*, *Runt*, *Krüppel-like,* and *GATA factors*, are activated during embryogenesis or regeneration in other animals. Furthermore, the genes involved in the specification of endoderm and the digestive system formation during the development of animals, such as, in particular, *Sox9*, *SoxD1* [106,107], *GATA4/5/6* [108], and a number of other ones, are expressed only in dividing individuals. The presence of a large number of transcripts of genes involved in the differentiation regulation and morphogenesis is the evidence that holothurian’s tissues begin preparing for the subsequent regeneration during fission.

Thus, analysis of *C. schmeltzii* transcriptome has shown that holothurians demonstrate a broad variety of structural components of connective tissue and proteins modifying it. Structural proteins such as collagens, fibrillins, and fibulins, as well as various proteases and their inhibitors, may be involved in ECM modification during fission. It is probable that a special group of TIMP-like proteins, tensilins, also plays certain role in this process. The fission process in holothurians is accompanied by significant physiological changes. The metabolism is increased, the nervous and immune systems are activated, and the structural changes occur in the fission zone. One of the significant indicators of morphological changes is the activation of the genes of laminins and nidogen, which probably an evidence of reorganization of epithelia of internal organs. To understand mechanisms of ECM remodeling, it would be probably necessary to pay more attention to the structure and functions of fibrillins in echinoderms. It is possible that the fibrillin microfibril scaffold, like that of vertebrates, forms a niche for regulatory factors and mechanosensation. During fission, a large number of factors, which probably regulate the division of body into parts and the preparation of tissues for subsequent regeneration, are expressed. The difference in the qualitative composition of the expressing TFs between intact and dividing holothurians will make it possible in the future to identify factors that regulate asexual reproduction. Moreover, the presence of transcripts of genes involved in regulation of morphogenesis of various tissues and organs indicate that the preparation of tissues for the subsequent regeneration in holothurian begins immediately during fission.

## 3. Early Events after Evisceration

In all studied holothurian species, the early events that occur in response to injury (cutaneous wound or evisceration) are approximately the same. The animals lose a coelomic fluid and a large number of coelomocytes that, in turn, causes noticeable stress and activation of protective functions. Damage and stress lead to blocking of many biological functions. A transcriptome analysis has shown that many genes related to signaling systems, cell adhesion, metabolic processes, and immune system are downregulated after evisceration [109]. The first holothurians’ responses to damage are aimed at restoring the cellular and protein composition of the coelomic fluid, activating the immune system, and healing the wound. 

One of the earliest processes registered in holothurians after injury is the expression of heat shock proteins (HSPs) [110]. In *Holothuria* (*Holothuria*) *tubulosa* Gmelin, 1791, the level of HSP90 in the coelomic fluid increases 5-fold within 1 h after the wound is inflicted [110]. According to Li et al. [111], HSP90 in humans is an extracellular tissue-repairing factor. Moreover, HSP90 can interact with MMPs and various receptors. During the same period, the level of HSP70 in damaged tissues of *H. tubulosa* increases almost 5-fold [110]. High HSP70 concentrations may probably trigger the cellular immune responses and increase the resistance to oxidative stress [112,113,114]. Subsequently, the level of these HSPs in the coelomic fluid and in the wound gradually decreases, but remains significantly higher within the first 2 days post-injury than in intact holothurians.

Also, one of the first responses to evisceration is the restoration of the number of coelomocytes. One hour after evisceration, the number of juvenile cells (the stem cells of coelomocytes) increases in the coelomic fluid of holothurians [26,27]. Among the cells, a subpopulation is identified that expresses the pluripotency marker *piwi*. The content of PIWI-positive cells was maximal at the initial stages of regeneration—1 and 4 h after evisceration. 24 h after evisceration, their number decreased as a result of differentiation of juvenile cells into amoebocytes and morula cells.

To protect themselves from invasion of pathogens, holothurians have components of both humoral and cellular immunity [115,116,117,118,119,120]. Immune cells of holothurians (coelomocytes) rapidly respond to various impacts. For example, when stimulated with bacterial lipopolysaccharides, they express a large number of genes associated with the immune response as early as within 4 h after exposure [120]. Within 24 h after cutaneous wound is applied, coelomocytes begin to express HSP27 and HSP70 [110]. HSP70 is probably involved in wound epithelialization. Furthermore, HSP70 continues to perform the role of innate immune system activator [112]. The role of HSP27 may be associated with the regulation of cell migration and differentiation, and also with protection from the wound-stressful environment [110]. Transcrips of *HSP70* are detected in gut anlagen of *A. japonicus* and *E. fraudatrix* [75,102].

As any damaging effect, evisceration causes oxidative stress manifested as an increase in the production of reactive oxygen species (ROS) [114,121]. The antioxidant enzyme system of cells, represented by superoxide dismutase, catalase, glutathione reductase, glutathione S-transferase, and glutathione peroxidase, is activated to provide protection against ROS [122]. Genes of the main enzymes of this system were identified in holothurians [102,119,123]. It was shown that the total antioxidant capacity in *A. japonicus* increases in the first 10 days post-evisceration and remains at a high level until the completion of regeneration [118]. At the same time, the dynamics of the activity of antioxidant enzymes varies between different tissues. It is worth noting that both the total antioxidant capacity and the superoxide dismutase activity increase in muscles that were not damaged by evisceration. This indicates that the removal of viscera has a stressful effect on the entire animal’s body and on all its tissues [118].

Stimulation of coelomocytes is also manifested as activation of their oxidant and antioxidant systems. ROS is known to play a significant role in phagocytosis. In vertebrates, generation of ROS during phagocytosis is balanced with a high antioxidant enzyme activity of phagocytes [124]. Thus, one of the indicators of holothurian immune system’s functioning is the activity of coelomocyte antioxidant enzymes [125]. It has been shown that amoebocytes (a type of coelomocytes that perform a phagocytic function) can produce ROS under various stress impacts [121]. However, they also show high levels of catalase, glutathione reductase, and glutathione S-transferase [126]. With various damaging effects, including cutaneous wound, the activity of these antioxidant enzymes in amoebocytes increases [127]. 

The response of humoral immunity is expressed as the synthesis and release of a large number of various factors, primarily lectins, in the coelomic fluid. Lectins are one of the major factors that protect holothurians from bacteria. In intact holothurians, many tissues, as well as coelomocytes, express C-type lectins [128,129,130]. Since transcripts of various lectins are found in transcriptomes of regenerating organs [71,102,116], it is likely that cells of anlagen can also synthesize these substances. 

The major role of lectins in the immune response is to interact with extracellular sugar residues that are part of the bacterial wall. It has been shown that C-lectin from *A. japonicus* (AjCTL-1) is a pattern recognition receptor [128]. Its expression is induced by gram-positive and gram-negative bacteria. AjCTL-1 can directly bind to peptidoglycan, agglutinate a broad spectrum of bacteria, and enhance the phagocytosis of coelomocytes. Moreover, a peptidoglycan recognition protein, AjPGRP-S, has recently been found in *A. japonicus* [131]. AjPGRP-S serves as a pattern recognition molecule involved in the inactivation of various bacteria [131]. The presence of lectins in intact tissues makes it possible to start inactivating bacteria immediately after damage without activating the corresponding genes. Endogenous reserves of lectins are gradually used up over the first three days after evisceration [129]. Probably only after this does the synthesis of new lectin molecules begin.

Thus, the early response to damage in holothurians is associated with the rapid release of protective molecules by various cells. This is necessary due to the fact that the stressful impacts block many biological functions, including the immune system [109]. Expression of putative immune-related genes such as *transferins*, *ferritin*, *serine protease inhibitor*, *α-2-macroglobulin*, *thymosin-β*, *C-type lectins*, *serum amiloid*, and *cathepsins* occur later, only after 3 days post injury [71,102,116,119]. Expression of some of them, e.g., *serum amyloid* and *melanotranspherins*, continues even in late stages of regeneration. This indicates their probable involvement not only in the immune response, but also in morphogenesis [115,132].

## 4. Regeneration after Posterior Evisceration

### 4.1. Morphological Aspects of Regeneration

**Figure 2:**
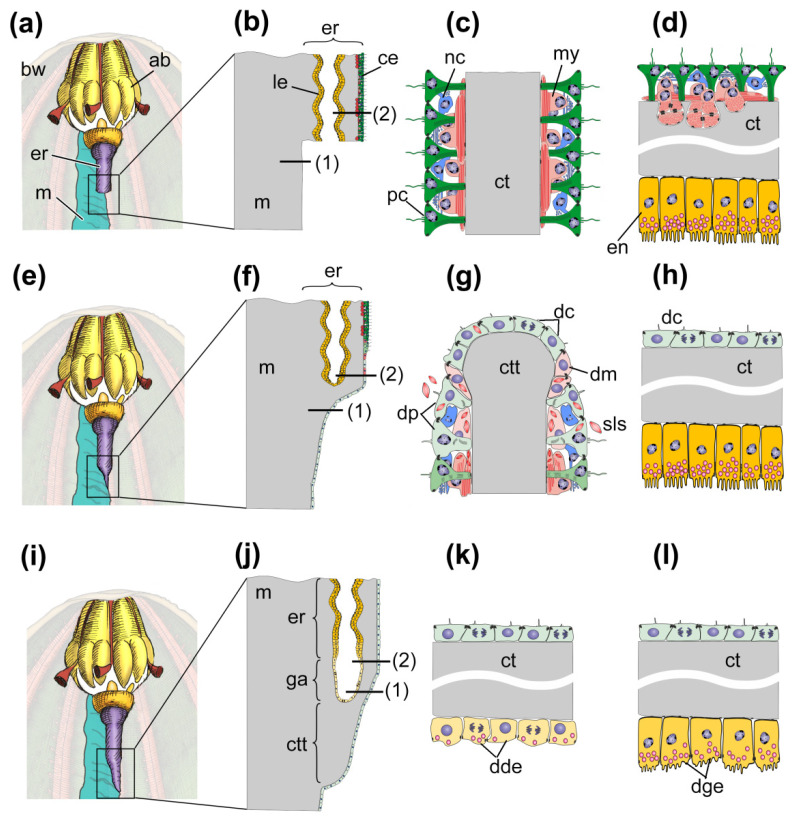
Scheme of regeneration of anterior part of digestive system after posterior evisceration. (**a**) Anterior part of holothurian just after evisceration; (**b**) Longitudinal section of the region of the esophagus remnant and mesentery, which is marked by the rectangle in (**a**). (**c**) Transverse section of the region of mesentery which is marked by solid line (1) in (**b**). (**d**) Transverse section of the region of esophagus remnant which is marked by solid line (2) in (**b**). (**e**) Anterior part of holothurian on third day post-evisceration; (**f**) Longitudinal section of the region of the esophagus remnant and connective tissue thickening, which is marked by the rectangle in (**e**). (**g**) Transverse section of the region of connective tissue thickening which is marked by solid line (1) in (**f**). (**h**) Transverse section of the region of esophagus remnant which is marked by solid line (2) in (**f**). (**i**) Anterior part of holothurian on seventh day post-evisceration; (**j**) Longitudinal section of the region of gut anlage, which is marked by the rectangle in (**i**). (**k**) Transverse section of the region of growing end of anterior gut anlage which is marked by solid line (1) in (**j**). (**l**) Transverse section of the region of growing end of anterior gut anlage which is marked by solid line (2) in (**j**). ab, aquapharyngeal bulb; bw, body wall; ce, coelomic epithelium; ct, connective tissue; ctt, connective tissue thickening; dc, de-differentiated coelomic epithelial cell; dde, de-differentiated enterocyte; dge, de-differentiating enterocyte; dm, de-differentiating myoepithelial cell; dp, de-differentiating peritoneal cell; en, enterocyte; er, esophagus remnant; ga, anterior gut anlage; le, luminal epithelium; m, mesentery; my, myoepithelial cell; nc, nerve cell; pc, peritoneal cell; sls, myofilaments grouped in spindle-like structures.

The processes of gut regeneration after posterior evisceration in *A. japonicus* and *H. glaberrima* are morphologically similar [12,37,41,62]. The digestive system formation is described in sufficient detail in *H. glaberrima* [37]; some data on the ultrastructure of gut anlagen are available for *A. japonicus* [24,41,62]. However, in a number of works, the interpretation of events occurring in one or another stage of regeneration is not entirely correct. For this reason, below I briefly describe the main stages of gut regeneration in these species (Figure 2).

#### 4.1.1. Initial Stage (1–24 h Post-Evisceration)

During evisceration, a significant amount of coelomic fluid and coelomocytes contained in it are lost. Furthermore, a rupture of the cloaca wall and, probably, the entry of pathogens into the body cavity occur in this stage. Thus, the first responses of the holothurians are aimed at restoring the volume of coelomic fluid and the number of coelomocytes, wound healing, activating the immune system, and cleansing the internal environment of microorganisms and damaged cells. There are no morphological changes in the damaged organs (Figure 2a–d).

#### 4.1.2. Preparation to Regeneration (1–3 Days Post-Evisceration, dpe) 

In this period, the first morphological signs of dedifferentiation of coelomic epithelial cells are observed (Figure 2e–h), the DNA synthesis begins in them [37]. During dedifferentiation, myoepithelial cells undergo the most pronounced changes. Myofilaments break down and spindle-like structures (SLSs) are formed in them; SLSs are then transported to the cell membrane and exocytized into the coelom (Figure 2g) [9,52]. The nerve cells of the basiepithelial nerve plexus disintegrate [12,133]. Within 2–3 dpe, connective-tissue thickenings begin to form at the posterior end of the esophagus remnant and at the cloaca along the torn edge of the mesentery (Figure 2e,f). These thickenings represent a connective-tissue framework or a basis for the future gut. Therefore, these thickenings are often referred to as “gut anlagen”. Subsequently, the thickenings extend along the mesentery edge towards each other.

#### 4.1.3. Onset of Regeneration (7–10 dpe) 

The main sign of the onset of this stage is the dedifferentiation of enterocytes in the cloaca and in the posterior part of the esophagus remnant (Figure 2i–l) [37,41]. The DNA-synthesizing activity of coelomic and luminal epithelium cells increases sharply [24,37,41]. In this case, however, the intercellular junctions are not destroyed, and the epithelium retains its integrity. After dedifferentiation, the esophageal and cloacal luminal epithelia begin to grow into the connective-tissue thickenings (Figure 2j).

#### 4.1.4. Regeneration and Growth of Gut (10–21 dpe) 

The formed anterior and posterior gut anlagen with enterocytes grow along the ventral edge of mesentery towards each other. They merge within 14–20 dpe. Luminal epithelium of the anlagen consists of dedifferentiated enterocytes that divide mitotically. Nevertheless, residues of secretory granules remain in their cytoplasm, i.e., no complete dedifferentiation occurs [24,41,62]. The cells of coelomic and luminal epithelia begin to differentiate at this stage. In coelomic epithelium, myoepithelial cells that form the gut musculature are found as early as after 10 dpe. Nerve fibers and neurons appear in coelomic epithelium after 14 dpe [133]. The enterocyte re-differentiation is observed in the same period. 

Thus, according to morphological data, the main regeneration processes in holothurians after posterior evisceration are dedifferentiation, cell proliferation, ECM remodeling, and epithelial morphogenesis. In *H. glaberrima*, some cells of coelomic epithelium migrate into the connective-tissue thickening [134]. However, these cells are not involved in formation the luminal epithelium, and the purpose of this process still remains unclear. It should be noted that, despite the proliferation, no blastema (as an aggregation of undifferentiated actively proliferating mesenchymal cells) is formed in holothurians during the gut regeneration. This point of view is confirmed by the fact that numerous studies of regenerating gut transcriptomes in *A. japonicus* and *H. glaberrima* have not revealed any expression of genes associated with the epithelial–mesenchymal transition [72,73,74,75,109].

### 4.2. Transcriptomic, Proteomic and Metabolomic Data

Many studies on transcriptomes, proteomes, and metabolomes with assessment of global processes occurring in regenerating tissues of the holothurians *A. japonicus* and *H. glaberrima* have been published to date. These are quite difficult to compare, since different tissues were analyzed. In *A. japonicus*, the remnant of the esophagus and the anterior gut anlage were examined; in *H. glaberrima*, both anlagen and the entire gut mesentery located between them were studied. Moreover, in *H. glaberrima*, the connective tissue thickenings which have not enterocytes were analyzed in many cases. Nevertheless, these studies provide the necessary basis for the further, more detailed analysis of events that take place on the molecular level during the digestive system regeneration in holothurians.

#### 4.2.1. 1–3 Days Post-Evisceration

The first signs of coelomic epithelial cell activation on the molecular level are detected as early as at 24 h post-evisceration. During this period, there is an increase in transcripts of genes involved in transcription, translation, and protein transport [109]. The activation of coelomic epithelial cells apparently consists in increased expression of genes encoding common transcription factors such as *TAF1A*, *MYBBP1A*, *PWP1*, and *EEF1A1* [109]. The maximum expression of all these genes is recorded at 1 dpe, and the expression decreases slightly after 3 dpe. This complements the morphological data on the early activation of coelomic epithelium cells in holothurians after damage [9,42,43,52].

After 3 dpe, these processes are also detected in the esophagus remnant [74,75,135,136,137]. An analysis of metabolites and phosphorylation and acetylation processes has shown that a significant rearrangement of cellular metabolism occurs in the holothurian esophagus after 3 dpe [135,136]. As in the activation of the mesenterial coelomic epithelium, it influences the key processes: Transcription, translation, and processing of proteins. All of these occur along with the ongoing stress and the immune system activity [115,118,129,132]. For example, during this period, the level of betaine in the esophagus remnant in *A. japonicus* rises, and the expression of *betaine-aldehyde dehydrogenase* increases [135]. Subsequently, the betaine concentration decreases. One of the betaine functions is to participate in osmoregulation [138]. It is likely that an increase in its concentration in the early period post-evisceration is associated with the loss of coelomic fluid and a disturbance of water-salt metabolism in holothurians.

Data obtained by a metabolomic analysis show that the processes indicating the accumulation of nutrients at the site of damage occur in tissues of the esophagus remnant after 3 dpe. Almost half of the proteins involved in the amino acid transport and metabolism were upregulated [137]. It is worth noting here that almost all proteins associated with the “energy production and conversion” term underwent deacetylation [136]. Thus, the accumulation of energy reserves and their further utilization during regeneration in holothurians is regulated by deacetylation [136]. The decrease in the lipid transport and metabolism is probably associated with energy accumulation [136,137]. In echinoderms, coelomic epithelium plays a key role in the accumulation and deposition of nutrients in the form of lipoprotein granules [139,140]. During the gut regeneration in the holothurian *C. schmeltzii*, lipoprotein granules are transported from coelomic epithelium to enterocytes in the early regeneration stages and are gradually utilized there [45,60]. All these processes are obviously one of the components of a strategy of energy relocation under various effects, which is common for all echinoderms [141,142].

During this period, the expression of genes associated with the transformation of the extracellular matrix and cytoskeleton increases in the mesentery, connective tissue thickenings and esophagus remnant. In particular, many ECM-associated genes (*collagens*, *laminins*, *MMPs*, *spondin*, and *fibropellins*) are up-regulated [72,75]. As already mentioned, the extracellular matrix transformation plays an important role in regeneration in holothurians. In the early period, the mesentery grows longer and changes its shape [62]. After 3 dpe, the mesentery edge begins thickening and forming connective-tissue gut anlagen at the cloaca and at the torn end of the esophagus (Figure 2e). All this requires intensifying the synthesis of connective-tissue components and their modification.

The expression of genes associated with the cytoskeleton transformation changes in different directions. Some of the genes were up-regulated; while others were down-regulated [72,75,109]. Furthermore, the phosphorylation and acetylation levels of most cytoskeletal proteins were shown to be up-regulated [135,136]. During this period, according to morphological data, coelomic epithelium undergoes major changes (Figure 2g). Thus, the cytoskeleton rearrangement is mainly associated with the dedifferentiation of coelomic epithelial cells. As mentioned above, the most pronounced changes are observed in myoepithelial cells. The complex process of their dedifferentiation is, undoubtedly, accompanied by a significant rearrangement of the cytoskeleton. The reported phosphorylation of neural alfa2 tubulin [136], obviously, indicates changes in the nerve cells of the basiepithelial nerve plexus of coelomic epithelium. A functional analysis of transcriptomes of the mesentery in *H. glaberrima* has shown down-regulation of the genes associated with cell adhesion such as, in particular, *cadherins* [109]. This is consistent with the migration activity of coelomic epithelial cells.

In the regeneration research, the genes involved in the regulation of morphogenesis such as TFs and components of signaling pathways are of great interest. Most TFs expressed in *H. glaberrima* within 1–3 dpe are associated with the development of mesodermal derivatives [109]. This is not surprising, because, as mentioned above, the holothurian mesentery is derived from the third pair of coelomes, somatocoels [56,57]. Orthologs of many of these TFs regulate various processes in vertebrates that occur in muscles, connective tissue, and skeletal elements. In many cases, they are involved in keeping cells in an immature state or inhibiting their differentiation. In particular, these are the *myc*, *Sox4* and *LMO2* genes. According to the heatmap of TFs presented in the work of Quispe-Parra et al. [109], all these genes show up-regulation after 1 dpe. It has been shown that down-regulation of *myc* decreases the radial glia dedifferentiation in *H. glaberrima* [143]. In mammals, *Sox4* maintains the undifferentiated state of cells [144], while *LMO2* is involved in hematopoiesis and dedifferentiation [145,146]. Thus, the activation of *myc*, *Sox4*, and *LMO2* as early as after 1 dpe indicates the early onset of coelomic epithelial cells dedifferentiation. 

A number of up-regulated genes were also identified in a study on the transcriptome of the esophagus remnant in *A. japonicus* at 3 dpe [75]. In particular, the expression of *Hox1* and *Hox3* was recorded to increase. The early activation of *Hox3* was also confirmed in the analysis of miRNAs [147]. The lack of *Hox1* and *Hox3* transcripts in the transcriptomes of *H. glaberrima* mesentery, probably, may indicate that these genes are expressed in holothurians only in the esophagus remnant. Simultaneously, the expression of the *HOX9/10* and *Hox11/13* genes is observed in *H. glaberrima* [72]. The main function of the *HOX* family genes is the formation of the anterior–posterior body axis in the development and regeneration of animals [148,149,150]. *Hox1* and *Hox3* are considered as “anterior” genes of this family and, respectively, are responsible for the development of the anterior parts of the body. Their activation in the remnant of *A. japonicus* esophagus in the early stages post-evisceration probably indicates the onset of determination of site where the anterior part of the gut will be formed. In *H. glaberrima*, the activation of the “posterior” members of the *HOX* family is, apparently, localized in the posterior part of the mesentery, which means the establishment of the posterior compartment of the digestive system formation.

In *H. glaberrima*, the expression of the *Wnt9* gene is recorded from the posterior anlage during this period [72,151]. The Wnt signaling pathway plays an important role in animal regeneration [152,153,154]. Furthermore, it can be involved in the formation of body axes in animals [152,155]. In *H. glaberrima*, the *Wnt9* gene and the signal cascade triggered by it in the posterior part of the mesentery, presumably together with *HOX* genes, are involved in establishing the posterior compartment of gut formation.

#### 4.2.2. 7 Days Post-Evisceration

The beginning of gut growth is accompanied by dedifferentiation of enterocytes and migration of luminal epithelium into the connective-tissue thickening (Figure 2i–l). In this regard, the increase in transcripts of genes associated with cell movement, *tubulins* and *actins*, is characteristic [75]. In *H. glaberrima*, this process was less pronounced when both anlagen and the mesentery were analyzed, but this species also showed a marked expression of these genes after 7 dpe [72].

At this stage, *Hox9/10* begins to be expressed in *A. japonicus* [75]. The detection of “posterior” members of the *HOX* cluster in the anterior anlage indicates the continuing process of marking the anterior–posterior axis in holothurians. The *Hox9/10* and *HOX11/13* transcripts still occur in the transcriptome of *H. glaberrima* [72].

A characteristic feature of the digestive system regeneration in holothurians is the activity of the Wnt signaling pathway. In *A. japonicus*, an increase in the number of *Wnt4* and *Wnt6* transcripts is recorded after 7 dpe [75]. In *H. glaberrima*, the *Wnt9* expression continues during this period [72]. Subsequently, transcripts of these genes are detected up to 14–21 dpe in both species. For this reason, this signaling pathway has been studied more in detail (see below).

#### 4.2.3. 14–21 Days Post-Evisceration

A transcriptome analysis has shown that in the late stages of gut regeneration and growth, the major groups of genes identified in the previous stages continue to be expressed, indicating that processes that started within 3–7 dpe are still functioning [72,75]. Indeed, judging by morphological data, the processes that occur within 14–21 dpe differ little in quality from those after 7 dpe. The main distinguishing feature of this stage is the re-differentiation of enterocytes.

The marking of the anterior–posterior axis in holothurians continues. In *A. japonicus*, the anterior anlage shows increased expression of the “posterior” *HOX* genes, *Hox9/10* and *Hox11/13*. In *H. glaberrima*, products of these genes, on the contrary, are not detected in the transcriptome after 14 dpe, but *Hox5* transcripts appear [72]. As in previous stages, the expression of genes associated with the ECM-remodeling continues [72,75,156]. This agrees with the morphological changes associated with the growth of the connective-tissue thickening and mesentery transformation.

A noteworthy feature of transcriptome data is the down-regulated expression of myogenesis-related genes, *myosins* and *gelsolin.* The content of transcripts of these genes after 7 dpe and in the subsequent stages of regeneration is lower than the normal one [72,75]. This contradicts the morphological data, according to which both holothurian species have myoepithelial cells with myofilaments already within 10–14 dpe [37,41]. The down-regulation of *gelsolin* can be explained by the fact that it can play a role of regulator and effector of apoptosis [157]. In this regard, its repression is required in the active processes of dedifferentiation, proliferation, and restructuring of the cytoskeleton. However, it should be taken into account that the “down-regulation” of *myosins* and *gelsolin* is manifested only when compared with the amount of transcripts of these genes present in intact gut. In fact, the products of these genes are present in anlagen as early as after 3 dpe [75]. The number of transcripts of *gelsolin* and some of *myosins* increases during regeneration. This indicates the myogenesis and synthesis of myosin filaments, with the activity of these processes being, probably, much lower than in intact gut.

In general, the transcriptome analysis confirms the main events of gut regeneration observed by morphological methods. There is a gradual decrease in the number of up-regulated differentially expressed genes during regeneration [72,75]. This indicates that the main processes (dedifferentiation, proliferation, and migration) are triggered in the early stages post-evisceration (3–7 dpe). It is worth mentioning that an increase in the Gene Ontology (GO) terms of biological function category is observed in *A. japonicus* over the course of regeneration (from 3 to 14 dpe) [75]. This probably indicates an increase in the diversity of processes that occur in the gut anlage. Indeed, according to morphological data, along with the ongoing migration of luminal epithelium, re-differentiation of coelomic epithelial cells, and growth of connective-tissue thickening within 10–14 dpe, enterocytes begin re-differentiation, the activity of genes along the anterior–posterior body axis varies, and the ECM composition changes. However, the transcriptome analysis has not allowed a reliable identification of the key genes associated with the regulation of the regenerative process and the dedifferentiation of coelomic and luminal epithelial cells.

### 4.3. Analysis of Expression of Single Genes

Transcriptomic data were used as a basis for further studies of events on the molecular level. The genes exhibiting differential activity during gut regeneration were studied more in detail. Since the main processes that occur during the gut tube formation in *H. glaberrima* and *A. japonicus* are ECM remodeling, cell dedifferentiation, cell migration, cell proliferation, and apoptosis, studies have been focused on them. Furthermore, much attention is paid to the mechanisms of morphogenesis regulation by various factors.

#### 4.3.1. ECM Remodeling

According to biochemical and morphological data, the ECM composition changes in connective-tissue thickening. This is manifested, first, as variation in the number of collagen fibrils. Since gut regeneration in holothurians is based on the luminal epithelium migration into the connective-tissue thickening, therefore, the presence of collagen is expected to inhibit the gut growth [158]. It has been shown that collagen degradation in the connective-tissue thickening of *H. glaberrima* occurs after 5–7 dpe [100,158].

It is likely that several protease classes are involved in the degradation of collagen fibrils. As mentioned above, holothurians have a wide range of enzymes that can degrade collagen [71]. For example, in *H. glaberrima* and *E. fraudatrix*, several proteases with gelatinase activity are found in the gut anlage [97,100]. In *A. japonicus*, two *MMPs* genes have been described, *ajMMP-2* and *ajMMP-16* [96]. Transcripts of these genes were not detected in intact gut. *ajMMP-2* and *ajMMP-16* began to be expressed at 1–2 h post-evisceration. The maximum number of their transcripts was observed at 6 h and 1 day, respectively. The proteinases were recorded from the esophagus remnant at 3 and 7 dpe. The distribution in tissues of these MMPs varied. ajMMP-2 is localized only in luminal epithelium, while ajMMP-16 in all esophagus tissues. The differences in the expression of *ajMMP-2* and *ajMMP-16* genes and the distribution of the proteins encoded by them indicate different functions of these proteinases during gut regeneration in holothurians. Since ajMMP-2 and ajMMP-16 are detected only at the initial stage of regeneration, they are probably involved in the degradation of esophagus ECM and dedifferentiation of coelomic epithelial cells and enterocytes. Furthermore, ajMMP-2 and ajMMP-16 presumably regulate the interaction between ECM components and growth factors through site-specific proteolysis of ECM proteins and other biological molecules [96]. According to Pasten et al. [94], some cysteine proteases, mainly cathepsins, may be involved in collagen degradation in *H. glaberrima*. 

The important role of collagen degradation was shown in experiments with blocking of protease activity. With 1,10-phenanthroline inhibition, the amount of collagen in the mesentery and gut anlage in holothurians did not decrease during regeneration, and the rate of the digestive system formation slowed down [97,100]. The use of a more specific MMP inhibitor, GM6001, resulted in a complete cessation of the regeneration of longitudinal muscle bands in *E. fraudatrix* [99]. On the other hand, the substances that make up the amorphous component of connective tissue, such as proteoglycans, are retained in the ECM throughout the regeneration period in *H. glaberrima*. This indicates that they are necessary for the regeneration process, or, at least, do not interfere with it [158].

#### 4.3.2. Cell Proliferation and Apoptosis

Morphological data show a quite intensive cell division in the gut anlage. However, studies with transcriptome analysis [72,73,74,75] do not specify genes closely related to cell cycle progression, in particular, genes of the *cyclins* and *cdcs* (*cell division cycle*) families. Nevertheless, the amount of transcripts of these genes in the anterior anlage transcriptomes is higher than in intact gut of *A. japonicus* [75]. Thus, transcriptomic data is consistent with morphological data and confirms the activation of gene pathways associated with cell proliferation. The triggering and modulating of the proliferative activity is likely to be carried out by the Wnt signaling pathway [159].

Regeneration requires a regulated interaction between cell proliferation and cell death. Apoptotic cells substantially affect the behavior of surrounding cells such as, in particular, phagocytosis and proliferation [160]. The interaction of apoptotic and living cells makes a significant contribution to various physiological processes that occur during tissue remodeling, regeneration, and morphogenesis. Apoptosis is also observed during the digestive system formation in holothurians. The greatest number of dying cells is detected in coelomic epithelium of *H. glaberrima* after 3–14 dpe [161]. Apoptosis here may be one of the mechanisms of cell proliferation activation [160]. Coelomocytes producing ROS can also be involved in this process [162,163]. As already mentioned, coelomocytes and tissues in holothurians can produce ROS and antioxidant enzymes from the first hours after damage until late regeneration stages [118,119,121,125,126,127]. Another way of apoptosis’ effect on proliferation is by activating the Wnt signaling system. Apoptotic cells have been shown to activate Wnt signaling during regeneration in *Hydra vulgaris* [164]. Furthermore, the level of apoptosis and cell viability is also influenced by the intensity of *myc* expression [160].

The *survivin* gene plays certain role in holothurian gut regeneration [161]. Survivin (also known as BIRC5) is an evolutionarily conserved eukaryotic protein that is essential for cell division and can inhibit cell death [165]. During gut regeneration in *H. glaberrima*, it is expressed in coelomic and luminal epithelia. A comparison of the apoptosis intensity with the content of *survivin* transcripts allows drawing a conclusion about the anti-apoptotic role for survivin in the regenerating coelomic epithelium of the holothurian gut [161].

#### 4.3.3. Wnt Signaling Pathway

As mentioned above, the transcriptome analysis showed the activation of the Wnt signaling pathway during gut regeneration in holothurians [72,73,74,75]. In *A. japonicus*, a large number of genes encoding components of this signaling pathway are expressed in the anterior anlage [75,166,167]. In particular, transcripts of the *Wnt4*, *Wnt6*, *Wnt7*, *Wnt8*, and *WntA* genes are detected. The expression of *Wnt7* and *Wnt8* becomes maximum after 1–3 dpe, and then decreases to the values in control. In contrast, the largest number of *Wnt6* transcripts is recorded at 7 dpe and is maintained at a high level throughout the regeneration period [168]. The *WntA* gene in the esophagus remnant is activated immediately after evisceration. The number of its transcripts remains approximately at the same level up to 7 dpe and increases after 14 dpe. The WntA protein is localized mainly in coelomic epithelium. Thus, different dynamics of ligand expression indicates differences in the functions of the signaling pathways they trigger.

Similar results of studies on localization of expression were obtained for *H. glaberrima* [151]. A differential activity of only one gene of the Wnt family, *Wnt9*, was observed in this species. Its transcripts were recorded only from coelomic epithelium of the mesentery and both gut anlagen.

Quite a substantial effort has been made to study the role of Wnt signaling in gut regeneration in the holothurian *H. glaberrima* [159]. Most experiments were carried out on mesentery and connective-tissue thickenings, while gut anlagen (i.e., connective-tissue thickenings containing the growing luminal epithelium) remained largely unstudied. Thus, in the studies conducted, the involvement of the Wnt signaling pathway in the transformation of coelomic epithelium and connective tissue was, in fact, clarified. It was shown that the main role of Wnt signaling is to regulate cell proliferation. This regulation, as in other animals, is probably carried out through apoptosis, ROS production, and *myc* expression [160,162,163]. In *H. glaberrima*, the suppression of *myc* via RNAi led to a decrease in cell proliferation [169]. Similar results were also obtained for *A. japonicus* [167]. When *Wnt7* and *dishevelled* were inhibited via RNAi, there was a significant slowdown in the growth of the gut anlage.

Thus, the Wnt signaling pathway plays an essential role in gut regeneration in holothurians. Apparently, its main function is to regulate the proliferative activity and apoptosis of coelomic epithelial cells. In *A. japonicus*, it involves *Wnt7* and the corresponding signaling cascade. The role of other ligands and signaling pathways triggered by them has not yet been identified for sure. Furthermore, the difference in the Wnt gene expression spectrum between *A. japonicus* and *H. glaberrima* still remains unclear.

It should be noted that the Wnt family proteins can trigger canonical and non-canonical signaling pathways [170]. The role of non-canonical pathways is to regulate cytoskeleton transformations, cell migration, and calcium metabolism [170,171,172]. Therefore, they may also be involved in gut regeneration in holothurians. It is also worth mentioning that the genes encoding the components of non-canonical signaling pathways are expressed in the anterior gut anlage in *A. japonicus* [167]. These are such genes as *Rho*, *DAAM*, *Calcineurin*, *Rac*, *Ncd*, *Jnk*, and *NFAT*.

#### 4.3.4. Retinoic Acid Signaling

Retinoic acid compounds play a substantial role in the regulation of various functions in animals, including regeneration [173,174,175,176]. They act through binding to a special family of nuclear receptors. Transcripts of orthologs of the retinol (RXR) and retinoic acid (RAR) receptor genes are found in holothurian transcriptomes [75,109]. In *H. glaberrima*, they have been studied more in detail [177,178]. Three isoforms of RAR and two of RXR have been identified [177]. The genes encoding these receptors are expressed in the mesenteries, respiratory trees, muscles, gonads, and the digestive tract. Moreover, two enzymes involved in retinoic acid metabolism have been identified: Dehydrogenase reductase 7 and aldehyde dehydrogenase 8A1 [178]. The expression of genes encoding both receptors and enzymes changes little during gut regeneration in *H. glaberrima*. However, blocking of retinoic acid receptor and aldehyde dehydrogenase led to a decrease in proliferative activity of coelomic epithelial cells, a reduction in the size of the connective tissue thickening, and inhibition of coelomic epithelial cells dedifferentiation. Thus, retinoic acid signaling pathway is involved in the regulation of proliferation and dedifferentiation of coelomic epithelium, as well as in ECM remodeling.

**Figure 3:**
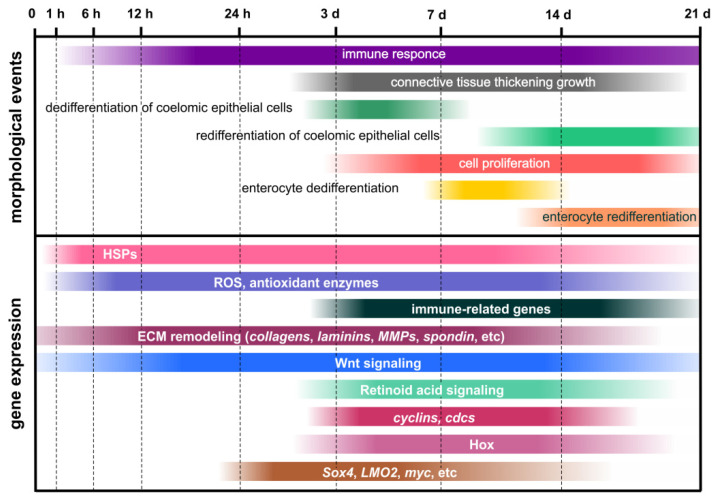
Schematic representation of morphological and molecular events during visceral regeneration after posterior evisceration. Timeline is represented by hours and days of regeneration after evisceration.

Thus, studies of single genes and signaling pathways in the holothurians *A. japonicus* and *H. glaberrima* have revealed some upstream regulators of the regenerative process (Figure 3). Wnt and Retinoic acid signaling pathways play an important role in the activation of coelomic epithelial cell proliferation and dedifferentiation. Apoptosis, which can occur with the involvement of ROS-producing cells, also makes a certain contribution to the triggering of cell division. In addition, Wnt and Retinoic acid signaling, probably, positively regulates ECM synthesis and growth of the connective-tissue thickening. The ECM remodeling involves various enzymes that degrade collagen and other connective-tissue components. Nevertheless, no genes involved in the transformation of luminal epithelia of the cloaca and the esophagus remnant during regeneration after posterior evisceration in holothurians have been identified to date.

## 5. Regeneration after Anterior Evisceration

### 5.1. Morphological Aspects of Regeneration

**Figure 4:**
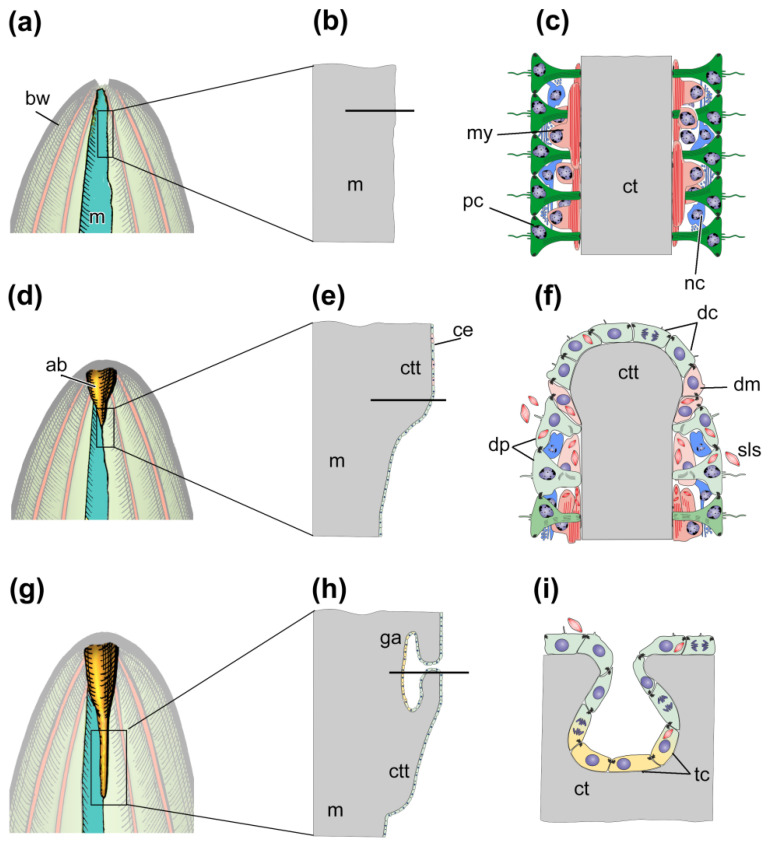
Scheme of regeneration of anterior part of digestive system after anterior evisceration. (**a**) Anterior part of holothurian just after evisceration; (**b**) Longitudinal section of the region of the mesentery, which is marked by the rectangle in (**a**). (**c**) Transverse section of the region of mesentery which is marked by solid line in (**b**). (**d**) Anterior part of holothurian on third day post-evisceration; (**e**) Longitudinal section of the region of the tissue thickening, which is marked by the rectangle in (**d**). (**f**) Transverse section of the region of connective tissue thickening which is marked by solid line in (**e**). (**g**) Anterior part of holothurian on seventh day post-evisceration; (**h**) Longitudinal section of the region of gut anlage, which is marked by the rectangle in (**g**). (**i**) Transverse section of the site of embedding of coelomic epithelial cells into connective tissue thickening which is marked by solid line in (**h**). ab, aquapharyngeal bulb; bw, body wall; ce, coelomic epithelium; ct, connective tissue; ctt, connective tissue thickening; dc, de-differentiated coelomic epithelial cell; dm, de-differentiating myoepithelial cell; dp, de-differentiating peritoneal cell; ga, anterior gut anlage; m, mesentery; my, myoepithelial cell; nc, nerve cell; pc, peritoneal cell; sls, myofilaments grouped into spindle-like structures, tc, coelomic epithelial cell during transdifferentiation.

The morphological aspects of gut regeneration after anterior evisceration have been described in sufficient detail only from one holothurian species, *E. fraudatrix* [24,42,43,46,61]. The early post-evisceration stage is approximately the same as in *A. japonicus* and *H. glaberrima* (Figure 4a–f). Within the first 3 dpe, the wound at the anterior end of the animal is healed [42], immunity is activated, the cell composition of the coelomic fluid is restored [26,27], and connective-tissue thickenings are formed along the mesentery edge (Figure 4d–f) [42,61]. The main difference from *A. japonicus* and *H. glaberrima* is that *E. fraudatrix* ejects not only the gut, but also the aquapharyngeal bulb (AB) during evisceration. As a result, regeneration begins with the formation of the AB anlage, and then, after 3 dpe, the anterior connective-tissue thickening begins to grow from it along the mesentery edge [42].

After 5–7 dpe, the coelomic epithelial cells are embedded into the connective-tissue thickening and begin transdifferentiation (Figure 4g–i) [46]. The embedding occurs in a quite limited area on the ventral side of mesentery, near the AB anlage. The embedded cells divide and gradually migrate anteriorly and posteriorly inside the thickening, forming the luminal epithelium of the anterior gut anlage. The cells do not lose relationships with each other, and intercellular junctions are maintained. Transdifferentiation proceeds quite quickly, and the signs of specialization of enterocytes in luminal epithelium appear after 10 dpe [46]. The posterior anlage forms in the same way as in *A. japonicus* and *H. glaberrima.* Subsequently, both anlagen of the gut grow along the mesentery edge towards each other and merge into a single digestive tube within 15–20 dpe.

### 5.2. Transcriptomic Data

A total of 11 genes of TFs were identified and characterized in *E. fraudatrix* that showed higher transcriptional activity during transdifferentiation (5–7 dpe) than in the previous and subsequent stages (3 dpe and 10 dpe, respectively) [102]. They belong to 6 TF classes: Tryptophan cluster (Ef-*elf*), C2H2 zinc finger (Ef-*prdm9*, Ef-*egr1*, Ef-*klf1/2/4* (*klf2*), Ef-*snai2*), bHLH (Ef-*tcf24*, Ef-*msc*, Ef-*id2*), C4 zinc finger (Ef-*gata3*), polycomb group ring finger (Ef-*pcgf2*), and T-box (Ef-*tbx20*). During this period, in addition to the embedding and transdifferentiation of a part of coelomic epithelial cells, the dedifferentiation and proliferation of coelomic epithelial cells on the surface of mesentery and anlage, and also the ECM synthesis and modification continue in the anterior gut anlage of *E. fraudatrix*. It can be assumed that genes whose expression increases during this period are involved in the regulation of one or more of the above mentioned processes.

The dedifferentiation, proliferation, and maintenance of the undifferentiated state of coelomic epithelial cells can involve *Ef-elf*, *Ef-prdm9*, *Ef-klf1/2/4*, and *Ef-egr1.* Ef-ELF belongs to the Ets family, whose members are powerful regulators of cell proliferation, angiogenesis, hematopoiesis, tumor transformation, and differentiation [179,180]. Proteins of the PRDM family are important epigenetic regulators of development, cell differentiation, and pluripotency in mice [181]. The KLF proteins play diverse roles in cell proliferation, differentiation, and development [182]. EGR1 is involved in the cell cycle progression of various tumor types, and also in hepatic regeneration in mammals [183,184].

The *Ef-gata3*, *Ef-egr1*, and *Ef-klf1/2/4* genes may be involved in the regulation of transdifferentiation. The *Ef-gata3* gene is of particular interest as its homolog in *Caenorhabditis elegans* is involved in transdifferentiation [185]. Moreover, it regulates the mesoderm and endoderm specification in echinoderms and mammals [186,187]. In planarians, EGR1 is a “putative pioneer factor to directly activate wound-induced genes” in whole-body regeneration [188]. KLF2 and KLF4 are key TFs that maintain a stem cell-like state and somatic cell reprogramming [189]. It is worth noting that in the holothurians *H. glaberrima* and *A. japonicus* the expression of *klf1/2/4* does not change during gut regeneration [28,75,109]. The different dynamics of *klf1/2/4* expression between these two species and *E. fraudatrix* confirms presence of different gut regeneration mechanisms in holothurians [58].

*Ef-elf* and *Ef-id2* can be involved in processes associated with the digestive system development and enterocyte differentiation. The expression of sea urchin *elf* is detected everywhere in late gastrula, being more concentrated in the gut [190]. The *id2* gene encodes a protein that, strictly speaking, is not a TF, since it lacks the basic DNA-binding domain. Nevertheless, it plays an important role in the digestive system development in vertebrates by preventing the precocious differentiation of the embryonic intestinal epithelium [191].

As is mentioned above, in the period when transdifferentiation occurs, the dedifferentiation of myoepithelial cells continues on the surface of the gut anlage. Among the identified TFs, *Ef-msc* and *Ef-tbx20* may probably be involved in the regulation of this process. In mammals, MSC (musculin) is a lineage-restricted repressor of embryonic skeletal muscle development [192]. TBX20 is a crucial cardiogenic TF in mammals [193]. The *tbx20* gene is also expressed in the nervous system and embryonic lateral mesoderm in mice [193,194].

Homologs of three TFs, *Ef-pcgf*, *Ef-snai2*, and *Ef-id2*, in vertebrates are involved in the mechanisms of the epithelial–mesenchymal transition (EMT). PCGF2 is a chromatin-modifying protein that inhibits EMT and negatively regulates stem cell-like properties [195,196]. SNAI proteins play a crucial role in EMT and in repressing the mesenchymal-epithelial transition, being important in embryonic and tumor development [197,198,199]. On the other hand, a complex of SNAI and ID2 inhibits EMT [200,201]. Co-expression of *Ef-snai2* and *Ef-id2* may indicate a partial involvement of the EMT mechanisms during the gut regeneration in *E. fraudatrix*. It is probable that, while embedding in the connective-tissue thickening, coelomic epithelial cells acquire some mesenchymal features [202,203,204].

Gene *tcf24* was first described in 2002 from humans as a paralog of the *tcf23* [205]. Since then, no information about its functions has been obtained. Activation of *Ef-tcf24* during regeneration in holothurians is the first description of its involvement in regeneration. The increase in the *Ef-tcf24* expression after 5-7 dpe in *E. fraudatrix* suggests participation of this gene in gut regeneration in holothurians.

Moreover, genes of Cyclins, Cdcs, Retinoic acid receptors and some other proteins participating in retinoic acid anabolism, are up-regulated during regeneration of anterior gut anlage in *E. fraudatrix* [102].

### 5.3. Wnt Signaling Pathway

The Wnt signaling pathway is activated in the regeneration of the organs of the anterior part of the body in *E. fraudatrix* (AB and the anterior part of the gut). Gene transcripts of four ligands (*wntA*, *wnt4*, *wnt6*, and *wnt16*) and three receptors (*frizzled1/2/7*, *frizzled4*, and *frizzled5/8*) have been identified in the AB and gut anlagen [206]. All of them exhibit different expression dynamics. According to preliminary data, transcripts of the studied genes are localized in coelomic epithelium and organs of the water–vascular system of forming AB [207].

The increase in the *wnt16* expression occurs earliest of all, after 3 dpe. In vertebrates, the *wnt16* expression is often associated with the development or regeneration of connective-tissue structures, the transformation of the extracellular matrix, and the activation of metalloproteinases [208,209,210]. In *E. fraudatrix*, an increase in the activity of MMPs and ECM remodeling is observed in this period [97].

After 5-7 dpe, the expression of *wntA*, *wnt4*, *wnt6*, *frizzled1/2/7*, and *frizzled4* increases abruptly and reaches its maximum. At this time, the main structures of AB and luminal epithelium are formed [42,46]. It is probable that Wnt signaling, performed through these ligands and receptors, influences the regeneration of the canals of the AB water–vascular system (water–vascular ring and radial water–vascular canals), and is also involved in the regulation of transdifferentiation. 

The following regeneration period (10 dpe) is characterized by either reduced or insignificantly changed expression of most of the studied genes. The exception is *frizzled5/8*, whose number of transcripts reaches its maximum. Subsequently, after 14 and 20 dpe, changes in the expression of all Wnt and frizzled genes are minimal. It is worth mentioning that the dynamics of *frizzled1/2/7* differ between *E. fraudatrix* and *A. japonicus* [167,206]. This may be explained by different functions of pathways which activated through the receptor Frizzled1/2/7.

Furthermore, the Wnt5 expression is revealed during the regeneration of the anterior structures in *E. fraudatrix* [211]. Unlike other ligands, Wnt5-positive cells were localized mainly in the connective tissue of the AB anlage and regenerating nerve cords. The number of such cells gradually increases after evisceration, reaching a maximum after 10-14 dpe, and then drops to the control values. The probable function of Wnt5 is the involvement in the regulation of ECM remodeling and nervous system regeneration. Wnt5 is known to be a regulator of neuronal organization and growth in planarians [212]. In mammals, Wnt5A is involved in the activation of MMPs during the development of skeletal elements [213].

### 5.4. Analysis of Expression of Single Genes

One *timp* (*tensilin*) and two *mmps*, upregulated during regeneration of the anterior organs, have been identified in *E. fraudatrix* [101]. The *timp* transcripts are detected in the gut anlage after 5 dpe. They are localized in a narrow band of cells on the ventral side of the mesentery and connective-tissue thickening. After 7 dpe, the expression pattern persists, but the number of *timp* transcripts near the AB anlage on the ventral side of the connective-tissue thickening decreases. This location roughly corresponds to the embedding zone of coelomic epithelium in ECM. After 10 dpe, the *timp* expression in the gut anlage is reduced. Throughout this period, *mmps* are diffusely expressed in the ventral part of mesentery and in the gut anlage. Thus, it is likely that the local *timp* repression determines the site where coelomic epithelium is to be embedded. By removing inhibition from MMPs, they enable cells to dissolve ECM and migrate into it.

Apparently, the following molecular events occur during the coelomic epithelium embedding. Initially, the connective-tissue base of the AB and anterior part of the gut develop. This connective-tissue thickening contains a large amount of collagen for stability. To maintain the anlage stability, the coelomic epithelium cells express TIMPs and, probably, other inhibitors that block protease activity and prevent the collagen degradation. After 5–7 dpe, local repression of *timp* caused by the activity of yet unknown factors is recorded. The decrease in *timp* expression removes the inhibition of MMPs and allows them to degrade collagen and modify ECM at this site. The reduction in collagen content is associated with the activation of coelomic epithelial cell migration [158]. Such a local change in the ECM composition probably triggers the processes of coelomic epithelium embedding in the connective-tissue thickening and transdifferentiation of its cells. 

The genes *sox9/10* and *sox17α* are apparently involved in transdifferentiation and further specification of enterocytes [101]. After 5–7 dpe, they are expressed in the coelomic epithelial cells on the surface of the anterior gut anlage. After 10 dpe, their transcripts are found in luminal epithelium of the anterior gut anlage. These results agree with data on other animals in which orthologs of these genes are involved in the endoderm specification and digestive system regeneration [106,107,214].

Genes of *HOX* family (*Hox1*, *Hox3*, *Hox5*, *Hox7*, *HOX 8*, *Hox9/10*, *Hox11/13a*, and *Hox11/13c*) are up-regulated during regeneration of anterior body part in *E. fraudatrix* [215]. Their expression begins to increase after 3 dpe and remains above control level throughout regeneration.

**Figure 5:**
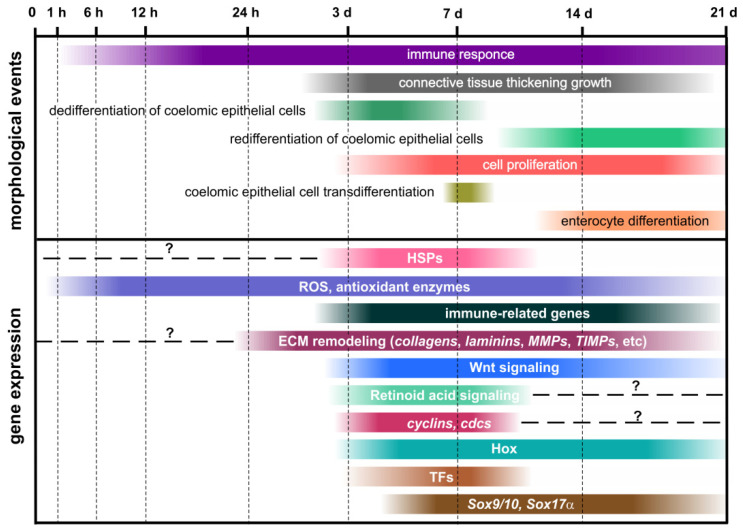
Schematic representation of morphological and molecular events during visceral regeneration after anterior evisceration. Timeline is represented by hours and days of regeneration after evisceration. Horizontal dotted line means no data.

Thus, studies of molecular events during regeneration in *E. fraudatrix* have revealed some upstream regulators of the regenerative process (Figure 5). Wnt signaling pathway play an important role in the activation of coelomic epithelial cell and AB formation. The ECM remodeling involves MMPs and TIMPs interaction. Among the identified TFs, *Ef-gata3*, *Ef-egr1*, and *Ef-klf1/2/4* deserve special attention as possible regulators of transdifferentiation. Other TFs, such as *sox9/10* and *sox17α* apparently participate in regulation of transdifferentiation and further specification of enterocytes.

## 6. Nervous System Regeneration

Another organ system in holothurians whose molecular aspects of regeneration are currently studied is the nervous system. In holothurians, it is represented by the nerve ring, located in the AB, and five radial nerve cords extending from it, which stretch along the inner surface of the body wall antero-posteriorly, up to the cloaca [56]. The regeneration of radial nerve cords after transverse cutting is described in sufficient detail on the morphological level from *E. fraudatrix* and *H. glaberrima* [39,216]. Some molecular aspects of nerve cord repair have been studied in *H. glaberrima* [28,30,143].

A transcriptome analysis has shown that, as in gut regeneration, the processes related to synthesis and organization of ECM components, ECM remodeling, and interaction between cells and extracellular matrix play a major role in the radial nerve cord repair [30]. This is not surprising, since the radial nerve cord in holothurians is located in the connective tissue of the body wall and, when it is cut, the body wall is also damaged (whole or partially, depending on the method of cutting). Many genes associated with normal physiology, differentiation, and development of the nervous system in *H. glaberrima* are down-regulated not only in the early stages (2 and 12 days post-injury), but also on day 20 post-injury. This is probably explained by the active dedifferentiation processes that occur during the nervous system regeneration in holothurians [30,39].

On days 2 and 12 post-injury, there is an increase in the expression of genes associated with the synthesis of DNA and proteins, and also with the cell cycle [30]. This correlates with morphological data on the activation of dedifferentiation and proliferation during this period [39]. Furthermore, the activation of genes associated with developmental processes is observed throughout the regeneration period. This indicates the involvement of ontogenic processes in the nervous system repair [30].

A total of 11 TF genes that show variation in expression during radial nerve cord regeneration were identified [30]. An increase in the number of transcripts at different stages after damage was recorded only for five of them: *NfkB1*, *serum response factor* (*SRF*), *liver X receptor*, *Fli1*, and *Esrrb*. Thus, the TFs are the most likely candidates for the role of regulators of nervous system regeneration in holothurians.

A transcriptome analysis revealed the *myc* gene expression during the radial nerve cord regeneration in *H. glaberrima* [30]. Further studies have shown that it is activated on day 2 post-injury and a high level of its expression is maintained throughout the regeneration period [28]. The cells expressing *myc* are found in apical regions of the neuroepithelia of the stumps, and also in glial tubes, which represent the early anlagen growing across the wound gap [28]. The presumptive role of Myc probably consists in regulation of the dedifferentiation of glial cells and programmed cell death [143].

## 7. Conclusions

The conducted analysis made it possible to identify common and distinguishing features of regeneration in different holothurian species. The greatest differences were observed between regeneration after fission and after evisceration. In case of asexual reproduction in *C. schmeltzii*, the damage and stress effects are minimal. Accordingly, the activation of the immune system and morphogenetic mechanisms occurs already during the transverse fission of the animal. The unique set of TFs that is expressed in *C. schmeltzii* indicates significant differences in regeneration mechanisms in asexual reproduction and after evisceration. 

Evisceration exerts a serious stress effect. The stress indicators such as increased ROS and activation of antioxidant enzymes are recorded within the first hours after removal of viscera and are detected even in the late stages of regeneration. Stress is also manifested as the inhibition of many biological processes in the early stages after evisceration. Protective immune responses are also triggered within the first hours after damage. The available data show that holothurians have a rather complex immune system represented by humoral and cellular components. Regeneration is activated along with the development of protective responses. The morphogenesis in holothurians occurs against the background of ongoing stress and immune activity. All three processes (stress, immune responses, and regeneration) are likely to be interrelated and can influence each other.

The early events associated with the preparation to regeneration are similar in *A. japonicus*, *H. glaberrima*, and *E. fraudatrix*. First, the cell composition of the coelomic fluid is restored. Then the activation of genes associated with coelomic epithelial cell dedifferentiation and ECM remodeling is recorded at 24 h post-injury. After that, the connective-tissue thickening begins to form in the anterior and posterior parts of holothurians. A feature common to all three species is also the timing of onset of the main regeneration stage—the formation of gut luminal epithelium. It starts after about 7 dpe. However, the mechanisms of luminal epithelium formation in *A. japonicus* and *H. glaberrima*, on the one hand, and *E. fraudatrix*, on the other, differ substantially on the molecular level, which is manifested as the expression of different gene sets. 

The ECM remodeling plays a major role in the regeneration in all the holothurian species under study. Connective tissue constitutes the basis for the formation of any organ. In echinoderms, it has specific features associated with the lack of elastin and a number of other components. This should be taken into account when analyzing the mechanisms of ECM functions during regeneration in these animals. One of the important regulators of ECM remodeling and all morphogenesis in holothurians is the Wnt signaling pathway. Due to the involvement of several ligands and receptors and their different spatio-temporal expression profiles, various processes related to dedifferentiation, proliferation, and apoptosis of cells of regenerating organs are fine-tuned. These processes involve also the retinoic acid pathway.

Coelomic epithelium plays an important role in the digestive system regeneration in holothurians. A wide range of genes associated with all of the above-mentioned processes are expressed in its cells. The Wnt signaling, which, together with the genes of the *HOX* cluster, apparently form an anterior-posterior gradient of gene expression determining the specification of morphogenetic processes along the anterior–posterior axis of the animal, is also activated in it. In case of regeneration after posterior evisceration, coelomic epithelium is not directly involved in the luminal epithelium formation, but may have an effect on ECM remodeling of gut anlagen and dedifferentiation of enterocytes. In *E. fraudatrix*, coelomic epithelium gives rise to luminal epithelium of the anterior part of the gut through transdifferentiation. A large number of TFs genes, which can be involved in the regulation of this process, are expressed in this case. The wide range of different genes activated in coelomic epithelium is the evidence of its important role in the morphogenesis regulation in holothurians. It is likely a key organizer of the entire regeneration process.

Despite a large number of transcriptomic, proteomic, metabolomic, and other molecular genetic studies, no molecular events leading to enterocyte dedifferentiation are known to date. It remains unclear what genes trigger and control this process. Furthermore, the obtained molecular data did not confirm the involvement of stem cells in regeneration in holothurians, nor were there any evident signs of increased expression of pluripotency factors have been identified, with the exception of the *myc* gene. The *myc* transcripts are detected in dedifferentiating cells. In holothurians, Myc is assumed to facilitate cell dedifferentiation and trigger the programmed cell death. 

In general, the array of available morphological and molecular data shows that the dedifferentiation of specialized cells of the organ remnant and the epithelial morphogenesis constitute the basis of regeneration in holothurians. Nevertheless, depending on the type of damage, the mechanisms of regeneration in holothurians may differ significantly in the spatial organization of regeneration process (one or two anlagen), the involvement of different cell types, and the depth of reprogramming of their genome (dedifferentiation or transdifferentiation). 

## Data Availability

Data sharing is not applicable to this article.
